# Paranasal sinus air suction for immediate pain relief of acute migraine – a randomized, double blind pilot study

**DOI:** 10.1186/s12883-019-1486-0

**Published:** 2019-10-23

**Authors:** S. M. R. Bandara, S. Samita, A. M. Kiridana, D. M. P. U. K. Ralapanawa, H. M. M. T. B. Herath

**Affiliations:** 1 Neurology Unit, Teaching Hospital Kandy, Kandy, Sri Lanka; 2 University of Peradeniya, Peradeniya, Sri Lanka; 3 Base Hospital, Matale, Sri Lanka; 4 Faculty of Medicine, University of Peradeniya, Peradeniya, Sri Lanka; 5 National Hospital of Sri Lanka, Colombo, Sri Lanka

**Keywords:** Migraine, Paranasal sinus nitric oxide, Para nasal air suction, aura, Pain relief, Nostril air flow rate

## Abstract

**Background:**

Migraine is a primary headache disorder, which cause significant disability in adolescence. This double blind, randomized clinical trial assessed the immediate effects of suction of paranasal sinus air during an acute migraine episode.

**Methods:**

A randomized, double blind study was conducted with 56 selected Sri Lankan school children of 16–19 years of age. Participants who met International Headache Society criteria for migraine (with or without aura) were included in the study. Subjects were randomly allocated into 2 groups where one group was subjected to three intermittent 10 sec paranasal air suctions with a ten sec suction free interval between two suctions for each nostril and the other group was subjected to placebo air suction (no paranasal air suction) in similar arrangement. Severity of headache and sub–orbital tenderness before and after suction were recorded using standard pain rating scale.

**Results:**

After dropouts, treated and placebo groups consisted of 27 and 23 subjects respectively. The mean headache pain score drop in the treated group was significantly higher compared to that of the control group. Moreover, there was a difference in the treatment response between the types of headache (with or without aura). With respect to tenderness there was a statistically significant drop in the treated group compared to the control. In general, airflow rates in left and right nostrils were different in these subjects. However such difference was not seen in the tenderness on two sides. Nevertheless it was revealed that airflow rate has a slight negative correlation with the tenderness irrespective of the side.

**Conclusion:**

Sixty–second paranasal air suction can provide an immediate pain relief for acute migraine in adolescents. We did not assess pain outcomes beyond 60 s, but the initial responses suggests the need to further study the efficacy of paranasal suction in migraine. A further study is suggested to evaluate the acute effects, efficacy and side effects of paranasal air suction using follow up over a prolong period.

**Trial registration:**

Sri Lanka Clinical Trials Registry SLCTR/2017/018, 29 Jun 2017. Retrospectively registered.

## Background

Migraine is a primary headache disorder and occurs at all ages. The estimated adult prevalence in Asia is around 3% in men and 10% in women, in USA 18% in women and 6% in men [1]. Split and Neuman stated that 28% of the adolescents had migraines with a female predominance [2] and migraine is the most common disabling primary headache disorder that occurs in children and adolescents. Chronic migraine is associated with missed school and poorer performance in school.

It is a condition characterized by recurring moderate to severe headache which is throbbing in nature that usually lasts from 4 h to 3 days, typically begins on one side of the head but may spread to both sides and is often accompanied with nausea, vomiting and sensitivity to light or sound. Sometimes it is preceded by an aura [3]. There are no specific neuroimaging findings associated with migraine in children and laboratory testing rarely is helpful. Migraine is the twenty first leading cause of disability–adjusted life years worldwide, sixth worldwide in the age group 25–39 years and tenth in Western Europe [4]. Adolescents with headaches have worse psychological functioning, more physical symptoms, poorer functional status, and less satisfaction with life and health than headache free controls [5, 6].Oral medications for acute migraine typically take more than 30 min to become effective [7]. In addition, electrical stimulation, magnetic therapy [8] and intranasal cryotherapy [9] have also been tried.

Many theories have been proposed to explain the patho–physiological mechanism of migraine, but no causative molecule for migraine has been identified [10]. Many molecules, rather than one molecule may be involved in migraine. One recent explanation is paranasal Sinus Hypoxic Nitric Oxide theory (SHNOT) for migraine, in which states that NO may play an important role in migraine. According to this hypothesis, diffused sinus nitric oxide (dsNO) in the nasal mucosa is hypothesized to be one of the main molecules involved in migraine patho–physiology [11]. In fact there is evidence for large concentration of NO in paranasal sinus cavities [12, 13]. It has been proven that neutralization of nasal NO after administration of intranasal NO scavengers can reduce migraine attacks and the severity [14] .

In migraine, there may be a partial or complete nasal obstruction of nostrils or ostial track due to parasympathetic over activity [15], anomalies of nasal and paranasal anatomy and polyps [16]. Obstruction to the airflow through nostrils and paranasal sinuses can be due to congestion caused by natural nasal cycle [17]. Therefore, suction of paranasal air mechanically can be used to reduce NO production as well as NO stagnation within the nasal and paranasal cavities. On this background, it can be assumed that acute migraine can be relieved by paranasal sinus air suction during an acute migraine attack. However this hypothesis has not been tested before and this study was aimed at assessing the immediate effects of nasal and paranasal air suction on pain relief in migraine.

## Methods

The study adheres to CONSORT guidelines. The type of design used was randomized, parallel–arm, double–blind, prospective study. The participants were selected from two stage randomization process with stage 1 being selection of schools randomly from Kandy district (an administrative unit) in Sri Lanka and stage 2 being selection of subjects randomly within the selected schools. A formal consent in writing was obtained from all participants who were 18 years of age or above and from parents or guardians of the participants who were below 18 years. The inclusion criteria were patients in the age group of 16–19 years, diagnosed with migraine according to the International Headache Society (IHS) criteria [3]. Patients who have not taken an acute treatment, at least for the testing period, with verification that they had more than 3 migraine attacks but not more than 15 attacks per month, were selected. Exclusion criteria considered were as follows; history of intracranial lesion or tumor, recent nasal or sinus infection, acute or chronic sinusitis, evidence of another infection (i.e., acute otitis media or pneumonia), history of allergic rhinitis, asthma or an underlying immune deficiency, cystic fibrosis, immotile cilia syndrome, recent head and facial trauma, runny nose, smoking, alcohol or drug abuse. Participants who were on hormonal therapy for any condition or illness, patients with psychiatric illness, patients on non–medical/non–nutritional treatment for migraine prevention such as acupuncture or psychotherapy, patients on fasting and had exercise or used any nasal drops or steam inhalation 1 h before the procedure and patients who did not consent were also excluded.

This was carried out as an outpatient study. When the selected participants presented with typical migraine attack (throbbing or pulsatile quality with nausea, vomiting, photophobia or phonophobia with or without aura) for more than 1 h they were randomized into treatment (paranasal suction) or placebo (placebo suction) group. All subjects were studied only once, during a single migraine headache. The primary endpoint was headache relief (defined as reduction of headache pain according to standard pain rating scale), immediately after the application of the device. The air sucker used had the following features; Compact High Suction Unit SUC81500, capacity of minimum of 32 L of free air per minute and suction pressure – 720 mmHg against a barometer of 760 mmHg. We adjusted the air suction pressure to 150 mmHg for the use in the study. A nasal tube attached to suction tube of the device was used for nasal air suction.

During the suction process, nasal and paranasal sinus air were sucked three consecutive times from each nostril. Each suction was for 10-s duration with a 10 s suction free period between two suctions. Thus each subject was subjected to 60-s suction altogether.

Test participants were instructed to hold the breath by closing both nostrils by his or her own hand. Then they opened one nostril for an application of the suction for 10 s. They were also instructed for mouth breathing if they need to breath during the suction period. After 10 s of suction free period, they closed the opened nostril and opened the other nostril for an application of air suction to that nostril for 10 s. During suction, the suction tube did not contact the skin or the mucosa of nostrils and was kept in the air space of the outer part of nostrils and very close to nasal orifice.

The control group was tested by keeping the same type of a nasal suction tube close to the nostrils. The appearance of the nasal air suction tube end of placebo group is similar to the test group. They were also asked to close and open the nostrils in a similar manner. However they were not exposed to air suction procedure though they were made to hear the sound of the air sucker. They got the instructions as the test group. All these measures were taken to provide similar perception to both groups to reduce placebo effect.

**Fig. 1:**
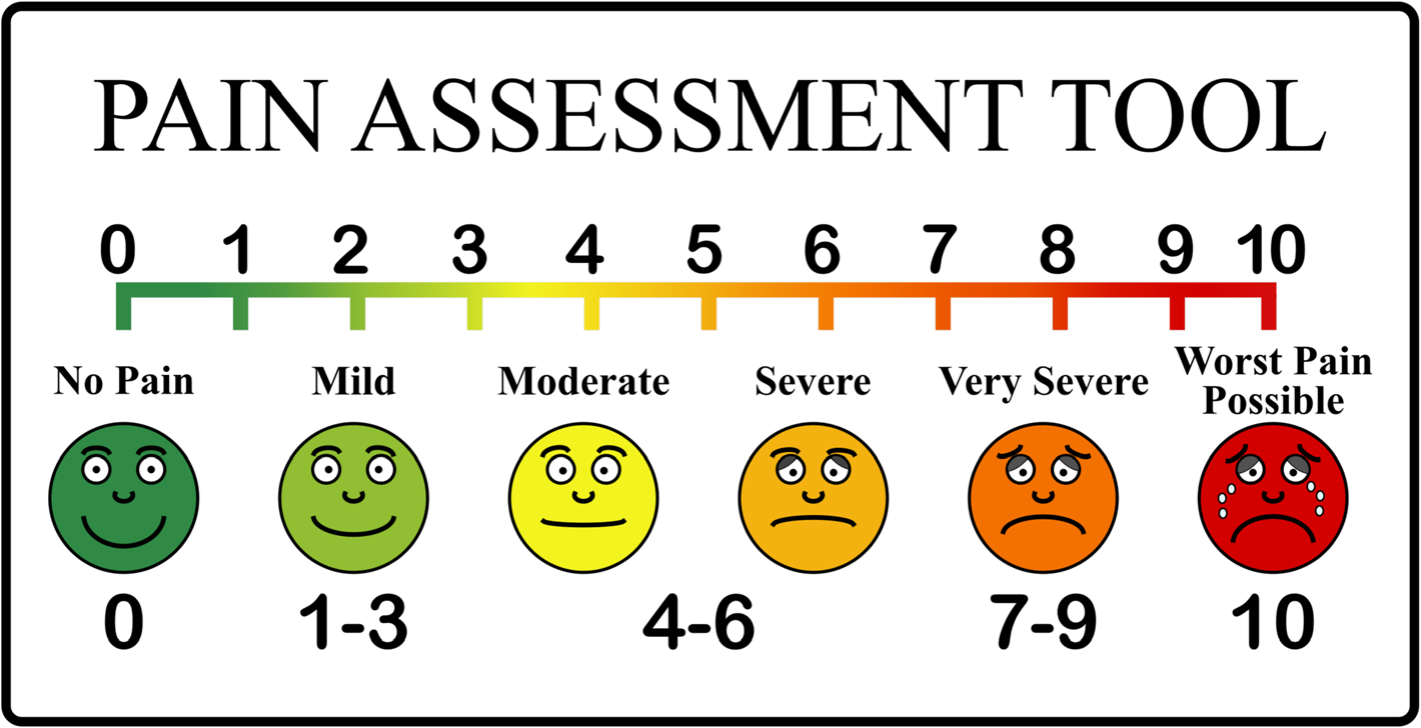
Pain rating scale used in the study

The severity of the migraine was measured using a standard pain rating scale (as shown in Fig. 1) with 0 being pain free and 10 being very severe pain before and after the air suction procedure. In addition to taking records on migraine pain, suborbital tenderness [18] was also recorded. When assessing tenderness over the scalp and suborbital area, the examiner applied pressure over the area until some blanching of their fingernail was discernible. The tenderness was assessed on both right and left suborbital areas. The severity of tenderness felt to the subjects was measured using the above pain rating scale. This tenderness was assessed both before and after the intervention. The nasal airflow rate of each nostril was also examined and documented. The investigator recorded the airflow rate on how he sensed the flow rate to second and third fingers as, none (0), mild (1), moderate (2) and normal (3). One investigator assessed this value during the whole study.

### Sample size

According to the study objectives, it was expected to detect a difference of 50 between the two groups with respect to mean score drop from before suction to after suction (mean score drop of 75 and 25 in the treated and placebo groups respectively). Since the pain scores are of ordinal nature, sample size formula for two sample non–parametric method given by

$$ n=\frac{{\left({Z}_{\alpha /2}+{Z}_{\beta}\right)}^2}{12c\left(1-c\right){\left(p"-\frac{1}{2}\right)}^2} $$

where

$$ p"=\frac{r"}{1+r"}, $$

$$ r"=\frac{P\left(Y>X\right)}{P\left(X>Y\right)}, $$

and c = 0.5 for equal sample sizes for both groups, was used to compute the required sample size. For a power of the test (1– *β*) of 0.8 and type I error rate (*α*) of 0.05, *Z*_*α*/2_ and *Z*_*β*_ are 1.96 and 0.84 respectively, and to detect a mean pain score drop difference of 50 (75 and 25), i.e., odds ratio of (0.75/0.25), required sample size was computed to be 21 for each group. To minimize the bias due to potential dropouts, a total of 56 subjects were selected, 28 per group.

### Statistical analysis

Descriptive statistical analysis outcome was used to describe the demographic factors of the study population. Since the response variable and the severity of pain was an ordinal measurement, non–parametric statistical methods were used to analyze the data. Specifically, in order to analyze the effect of air suction on drop of the pain score between before and after the intervention in different type of migraine (with and without the presence of aura) and side of headache (left, right or both), non–parametric method (Wilcoxon rank sum test) was used. SAS university edition software package was used for statistical analysis.

## Results

We screened 120 patients with migraine symptoms for eligibility from July 2017 to June 2018. Only 56 of them met clinical criteria for the diagnosis of migraine. Fifty-six patents were randomly allocated into two groups, 28 each and 6 dropouts occurred, one in the treated group and five in the placebo group. Ultimately twenty-nine male patients and twenty-one female patients participated in the study. The distribution of subjects under different factor levels is presented in Table 1.

**Table 1:** Distribution of subjects within gender and status of aura

Group	Male		Female	
	With aura	Without aura	With aura	Without aura
Treated	04	12	02	09
Control	02	11	04	06

The means of the drop in the pain score and sinus tenderness before the intervention and after the intervention are presented in Table 2. Table 3 contains the corresponding values under two types of migraine (migraine with and without aura).

**Table 2:** Means of the drop of the pain score and sinus tenderess from beginning to the end in treated and control groups for various measurements

Measurement	Treated	Control	P^*^
Pain	37.0	12.0	< 0.01
Left tenderness	36.6	12.5	< 0.01
Right tenderness	37.0	12.0	< 0.01

P^*^significant probability for the difference between two groups

**Table 3:** Means of the drop of the pain and sinus tenderness score from beginning to the end in treated and control groups in different types of migraines

Measurement	Status of aura	Treated	Control	P^*^
Pain	with aura	10.5	04.0	< 0.01
	without aura	27.0	08.5	< 0.01
Left tenderness	with aura	10.5	04.0	< 0.01
	without aura	26.6	09.0	< 0.01
Right tenderness	with aura	10.5	04.0	< 0.01
	without aura	27.0	08.5	< 0.01

P^*^significant probability for the difference between two groups

According to Table 2, the mean drop of pain in the treated and the control groups were 37 and 12 respectively and the pain drop in the treated group was much higher than that of the control group (*P* < 0.01). The impact of the treatment had varied depending on the presence and absence of aura. In patients with aura, the mean drop in the score in treated and the control groups were 10.5 and 4.0 respectively while these two figures were 27.0 and 8.5 in the absence of aura (Table 3). The mean drop in tenderness on the left side was 36.6 in the treated group as compared to 12.5 in the control group indicating a significant (*P* < 0.01) higher drop in tenderness in the treated group. Drop of tenderness on the right side was similar to that of left side and the mean drop in scores were 37 and 12 (Table 2) in the treated and the control groups respectively (P < 0.01). As with pain, the drop in the tenderness (on both sides) was higher in the absence of aura compared to in the presence of aura (Table 3). In this study almost all patients had sub–orbital tenderness (98% left side and 100% right side).

Outcome from the analysis on interaction between side of headache and type of intervention is presented in Table 4. According to Table 4 when headache is present on both sides, mean drop with respect to each of the three scores was much higher (P < 0.01) in the treated group as compared to the control group. However, when headache was present only on one side (left or right) this difference was not revealed purely because of the small number of subjects in such situations in the study. Nevertheless in the present context, it seems that an interaction is present between side of headache and the response to intervention, where the drop is higher in treated group when headache is present in both sides as compared to only on one side.

**Table 4:** Means of score drop from the beginning to the end in treated and control groups for recorded measurements classified by side of headache

Measurement	Side of headache	Treated	Control	P^**^
Pain	left	03.5 (4)^*^	01.0 (1)	0.35
	right	05.5 (8)	01.0 (1)	0.15
	both	29.0 (15)	11.0 (21)	< 0.01
Left tenderness	left	03.5 (4)	01.0 (1)	0.35
	right	05.4 (8)	01.5 (1)	0.27
	both	29.0 (15)	11.0 (21)	< 0.01
Right tenderness	left	03.5 (4)	01.0 (1)	0.35
	right	05.5 (8)	01.0 (1)	0.19
	both	29.0 (15)	11.0 (21)	< 0.01

^*^Values in parenthesis are number of subjects for the group

^**^significant probability for the difference between two groups

The nasal flow on both sides is present in the Table 5 and the analysis using Fisher’s exact test revealed that there is an association between the two variables (*P* = 0.01). The association is of the form that when one side airflow is low there is a tendency to have other side flow to be more or less normal. The Spearman correlation coefficient between the two variables was − 0.3409 (*P* = 0.02) and it confirmed the outcome from Fisher’s exact test that for a given individual when one side flow is poor, other side flow is more or less normal. After analyzing frequency distribution of the airflow rate score difference between two sides are given in Table 6. Pearson’s chi–squared test analysis of Table 6 showed that frequencies are different between scores in Table 6. From the sign rank analysis of air flow score differences (absolute values) of two sides, it was found that the mean difference was 1.22 with *P* < 0.01 and this also indicates that air flow on two sides are different for a given person. In addition only 9 subjects had no difference in the airflow on both sides and all other subjects there had been a different.

**Table 5:** Cross classification of nasal air flow (left) by nasal air flow (right) scores

Nasal air flow (left) score	Nasal air flow (right) score			
	0	1	2	3
0 (no flow)	0	0	4	0
1 (partial)	2	4	8	3
2 (moderate)	2	8	4	4
3 (normal)	1	9	0	1

**Table 6:** Frequency distribution of score differences of air flow between two sides

Score difference	Frequency
0	09
1	22
2	18
3	01

Cross-classification of tenderness analysis of two sides are presented in Table 7. Fisher’s exact test revealed that there is no association between the two variables (*P* = 0.58) and thus the severity of tenderness on two sides are independent of each other. The Spearman correlation analysis gave a correlation coefficient of 0.1736 (*P* = 0.23) and there by too it is shown that there is no pattern between the severities of tenderness on two sides.

**Table 7:** Cross classification of tenderness (left) by tenderness (right) scores

Tenderness (left) score	Tenderness (right) score				
	2	4	6	8	10
0	0	0	0	1	0
4	0	0	0	2	0
6	0	0	1	7	2
8	1	1	5	6	3
9	0	0	0	0	1
10	0	0	3	9	8

Spearman Correlation coefficients between air flow rate variables and tenderness variables are given in Table 8. According to Table 8, it is not possible to detect one to one correspondence between airflow and the tenderness of the same side. However, Table 8 indicates airflow rate is related to tenderness. In fact when both side values were pooled it was possible to detect a mild correlation between airflow rate and tenderness. The Spearman correlation coefficient was − 0.2076 (*P* = 0.04) and thus it indicates that there could be a slight trend on low airflow leading to high tenderness. However, since the correlation is mild, it is possible that a substantial fraction is having tenderness with normal airflow.

**Table 8:** Correlation matrix between air flow variables and tenderness variables

	Tenderness^a^ (left)	Tenderness^a^ (right)	Air flow (left)	Air flow (right)
Tenderness (left)	1.0000	0.1736 (0.23)^b^	−0.1178 (0.42)	0.1937 (0.18)
Tenderness (right)	0.1736 (0.23)	1.0000	0.3202 (0.02)	−0.3095 (0.03)
Air flow (left)	−0.1178 (0.42)	0.3202 (0.02)	1.0000	−0.3409 (0.02)
Air flow (right)	0.1937 (0.18)	−0.3095 (0.03)	−0.3409 (0.02)	1.0000

^a^Tenderness values are before intervention values

^b^Values in parenthesis are significant levels

## Discussion

Our main aim was to evaluate the effect of paranasal sinus air suction for immediate relief of migraine headache. It was evident that pain drop in the treated group is much higher than that of the control group (*P* < 0.01). This indicates paranasal air suction might be removing causative molecules in paranasal air and reduce or prevent excess production of NO or other air molecules, which can lead to acute relief of pain.

In this research it was found that suborbital tenderness also significantly dropped in the treated group compared to the control group. In addition it was found that almost all patients had suborbital tenderness (98% - left side and 100% - right side). Therefore this examination finding can be used as a symptom of migraine headache [18]. However the number of subjects in this research was small and so more research is needed to confirm this finding before using this as a diagnostic tool of migraine.

The interaction between the type of headache and the intervention could also be useful in migraine management. According to the study, the impact on the benefit of the treatment is higher when the aura is absent. Higher pain reduction in the absence of aura compared to in the presence of aura could be related to pathology of migraine. According to SHNOT, migraine with aura is brought about by more diffusion of NO through the mucosa of the upper respiratory track in the nasal cavity. Therefore in theory, migraine with aura there can be increased production of NO or/and other vasoactive molecules, increased absorptive surface area and more out flow obstructive anomalies in the ostial tack and nasal cavity compared to migraine without aura. Therefore it can be suggested that for migraine patients with aura nasal air suction duration can be extended and thereby reduce the availability of neuro–active air molecules in the vulnerable area. In fact this management is so important in the management of migraine with aura because it has a risk of stroke and other complications [19].

Paranasal air suction can be used to differentiate sinusitis from migraine because in migraine patients the pain and the tenderness can be relieved by nasal air suction procedure. In this research the headache subsided after 1 min of paranasal sinus air suction without any other treatment, which is not expected in sinusitis, and other secondary causes of headache.

Because of the significant reduction in headache and tenderness after paranasal sinus air suction, it is possible to assume that pathological process of migraine had subsided by air suction process. We can assume that suction of air has removed or reduced the synthesis of the neuro and vasoactive air molecules that might be the causative agents for migraine etiology. In fact it has been found that the potent neuronal stimulus, NO, is removed by nasal air blow with humming [20]. Therefore the beneficial effects of the application of nasal air suction can be due to removal of excess NO, which could be the causative and responsible molecule for migraine. Autonomic disturbance [21], release of calcitonin-gene related peptide at dural vessels with subsequent neurogenic inflammation [22], release of vasoactive intestinal polypeptide and pituitary adenylate cyclase-activating polypeptide by parasympathetic fibres innervating the dura mater [23] are also considered in migraine pathogenesis. Paranasal air suction may also remove these vasoactive substances involved in migraine pathology and reduce the headache and migraine.

According to airflow rate analysis, there is no one to one correspondence between particular side airflow rate and tenderness on the same side. However, the negative correlation between airflow and tenderness indicates that tenderness is affected by the airflow obstruction. This further proves the hypothesis of SHNOT for migraine that explains the excess nitric oxide absorption and stagnation caused by the out flow obstruction as a part of migraine pathology. Thus ventilation of sinus cavity by mechanical interference could be a strategy to reduce the nitric oxide absorption and stagnation. On the other hand, natural nasal cycle can also cause intermittent periodic nasal obstruction alternatively on two sides and thereby accumulation of NO leads to migraine. Nevertheless, the correlation is mild and thus it indicates that it is possible to have tenderness without having any airflow obstructions. In fact, the main cause of the airflow obstruction is inferior turbinate but excess production of vasoactive molecule in the paranasal area is still possible with normal inferior turbinates in patients with ostial tract obstruction or middle turbinate pathology.

The most important finding in the study is the advantage of nasal air suction to give rapid recovery from pain of migraine headache. Even though we did not assess the long-term and general safety of this procedure objectively, all the patients tolerated 60-s suction procedure without any side effects or complications.

### Limitations

This study is conducted as a pilot study and we did not evaluate the long term side effects of the nasal air suction. Even though the suction device we used is easy to use, it is larger and not easily portable. This limits the use of this device for acute migraine attacks since this is not available in every ward. However this provide us a basis to use suction device in acute migraine attacks which can be used in future studies using portable low grade air pressure suction device. We did not evaluate the neuro and vasoactive air molecules in the exhaust samples of paranasal air either. We only used a single measurement of both pain intensity drop and tenderness over sub – orbital sinuses immediately after the application of nasal air – suction and thus we may not be able to make conclusions on long term effects as well as on using this as an acute treatment. However this study has clearly indicated that immediate pain relief can be obtained by paranasal air suction and need further studies in order to determine whether this approach can be used as an acute treatment for migraine management. A second study is undergoing to evaluate the safety, side effects and efficacy of a portable low grade air pressure suction device that can be used in a day to day life, adverse effects of paranasal air suction procedure and to assess how long beneficial effects/pain relief last after air suction procedure using multiple measurements over 24 h.

## Conclusion

This is pilot study, which showed that paranasal air suction gave considerable immediate benefits in acute migraine in adolescents. 60-s nasal air suction can provide an immediate relief from migraine pain as well as reduce migraine induced suborbital tenderness. A further study is suggested to compare and evaluate the acute effect, efficacy and side effects of nasal air suction using multiple measurements over a prolong period.

## Data Availability

The datasets supporting the conclusions of this article are included within the article.

## Abbreviations

dsNO: Diffused sinus nitric oxide
NO: Nitric oxide
SHNOT: Paranasal sinus hypoxic Nitric Oxide theory
